# Different effects of cardiac and diaphragm function assessed by ultrasound on extubation outcomes in difficult-to-wean patients: a cohort study

**DOI:** 10.1186/s12890-017-0501-8

**Published:** 2017-12-01

**Authors:** Ling Luo, Yidan Li, Xiukai Chen, Bing Sun, Wenxiong Li, Wei Gu, Shuo Wang, Song Zhao, Yanwei Lv, Mulei Chen, Jingen Xia, Feng Sui, Xue Mei, Huanzhong Shi, Zhaohui Tong

**Affiliations:** 1 Department of Respiratory and Critical Care Medicine, Beijing Institute of Respiratory Medicine, Beijing Chaoyang Hospital, Capital Medical University, NO. 8 Gongren Tiyuchang Nanlu, Chaoyang District, Beijing, 100020 China; 2 Department of Respiratory and Critical Care Medicine, Beijing Jishuitan Hospital, NO. 31 Xinjiekou East District, Beijing, 100035 China; 3 Department of Ultrasound, Beijing Chaoyang Hospital, Capital Medical University, NO. 8 Gongren Tiyuchang Nanlu, Chaoyang District, Beijing, 100020 China; 4 Department of Surgery Intensive Care Unit, Beijing Chaoyang Hospital, Capital Medical University, NO. 8 Gongren Tiyuchang Nanlu, Chaoyang District, Beijing, 100020 China; 5 Department of Emergency Medicine, Beijing Chaoyang Hospital, Capital Medical University, NO. 8 Gongren Tiyuchang Nanlu, Chaoyang District, Beijing, 100020 China; 6 Department of Clinical Epidemiology Research Center, Beijing Jishuitan Hospital, NO. 31 Xinjiekou East District, Beijing, 100035 China; 7 Department of Cardiology, Beijing Chaoyang Hospital, Capital Medical University, NO. 8 Gongren Tiyuchang Nanlu, Chaoyang District, Beijing, 100020 China; 8 Department of Intensive care medicine, China-Japan Friendship Hospital, No.2 Yinghua East Street, Beijing, 100029 China

**Keywords:** Weaning, Extubation, Cardiac function, Echocardiography, Diaphragm function

## Abstract

**Background:**

Ultrasound is a convenient tool to evaluate cardiac and diaphragm function. The ratio (E/Ea) of mitral Doppler inflow velocity to annular tissue Doppler wave velocity by transthoracic echocardiography (TTE) and diaphragmatic excursion (DE) by diaphragm ultrasound have been confirmed in predicting extubation outcomes independently, however their different roles in the weaning process have not been determined until now.

**Methods:**

We designed a cohort study to preform diaphragm ultrasound and TTE before and after the spontaneous breathing trial (SBT) in difficult-to-wean patients. Patients considered for enrollment should succeed on a SBT and have been extubated. They were followed up with the events of respiratory failure within 48 h, and divided into the respiratory failure and extubation success subgroups. Relevant risk factors predicting respiratory failure were analysed by a multivariate logistic regression model. Then, each subgroup was assessed with respect to re-intubation within 1 week, and divided into the re-intubation and non-intubation subgroups. Furthermore, relevant risk factors predicting re-intubation were also analysed in each subgroup. The area under the curve (AUC) and optimum cut-off value were identified by the receiver operating characteristic curve.

**Results:**

Among 60 patients, 29 cases developed respiratory failure within 48 h, and 14 cases were re-intubated or died within 1 week, respectively. Multivariate logistic regression analysis showed that E/Ea (average) after SBT [odds ratio (OR) 1.450, 95% confidence intervals (CI) 1.092-1.926, *P* = 0.01] and left ventricular ejection fraction were associated with respiratory failure. The AUC of E/Ea (average) after SBT was 0.789, and a cut-off value ≥ 12.5 showed the highest diagnostic accuracy with a sensitivity and specificity of 72.4% and 77.4%, respectively. Furthermore, in the respiratory failure subgroup only DE (average) after SBT was associated with re-intubation (OR 0.690, CI 0.499-0.953, *P* = 0.024). The AUC of DE (average) after SBT was 0.805, and a cut-off value ≤ 12.6 mm showed the highest diagnostic accuracy with a sensitivity and specificity of 80% and 68.4%, respectively.

**Conclusions:**

E/Ea (average) after SBT could help predict respiratory failure within 48 h. However, DE (average) after SBT could help predict re-intubation within 1 week in the respiratory failure subgroup.

**Electronic supplementary material:**

The online version of this article (10.1186/s12890-017-0501-8) contains supplementary material, which is available to authorized users.

## Background

Predictors of extubation outcomes are consistently difficult issues for patients with a high risk of difficult weaning. Common reasons for extubation failure include primary respiratory failure, cardiac dysfunction, ineffective cough, excess secretions, upper airway obstruction and other aetiologies [1, 2]. The ideal indicators would not only predict extubation failure, but would also confirm the reasons for such failures. Traditional indicators, such as respiratory rate (RR), minute ventilation, tidal volume (V_T_), and the rapid shallow breathing index (RSBI), can reflect patients’ integral conditions, but it is difficult to apply them in the analysis of the exact reasons for such failures. Some studies have reported that B-type natriuretic peptide (BNP) or N-terminal-pro-BNP (NT-pro-BNP) could help identify patients who failed to wean due to cardiac dysfunction. Nevertheless, it was difficult to confirm the cut-off values; because they were easily affected by age, renal function, weight, infection, etc. [3–5].

Bedside ultrasound is a convenient tool to evaluate cardiac function, diaphragm function, lung aeration, etc. Cardiac dysfunction is one of the important risk factors for extubation failure. Recent studies have indicated that the ratio (E/Ea) of mitral Doppler inflow velocity (E) to annular tissue Doppler wave velocity (Ea) by transthoracic echocardiography (TTE) could help identify the cardiac origin of weaning failure [6–9]. In addition to cardiac dysfunction, diaphragm dysfunction is another critical reason for extubation failure. Some studies have reported that diaphragmatic excursion (DE) or liver/spleen movements could predict extubation failure [10–12].

Although TTE and diaphragm ultrasound have been confirmed in independently assessing extubation outcomes, few studies have shown their different roles in the weaning process until now. This observational study was designed to test the ability of cardiac and diaphragm function assessed by bedside ultrasound to predict respiratory failure within 48 h and re-intubation within 1 week after extubation.

## Methods

### Patients

This study was conducted in Beijing Chaoyang Hospital, which has a total of 58 beds in the 4 intensive care units (ICUs) (RICU, SICU, CCU and EICU), over a 16-month period (November 2012 to February 2014). The study was approved by an Institutional Review Board of Beijing Chaoyang Hospital on Sep 24th, 2012 (No.2012-ke-88). Consent forms were obtained from each patient’s immediate family members.

All patients were assessed for readiness to wean by their attending physicians. Once the patients failed the spontaneous breathing trial (SBT) at least once and had been intubated for more than 48 h, they were defined as difficult-to-wean patients [13]. Patients considered for enrollment should succeed on a SBT and have been extubated. The exclusion criteria were as follows: (a) pregnant women; (b) age < 18 years; (c) tracheostomy after the SBT; (d) severe mitral stenosis or prosthetic mitral valve; (e) a history of diaphragmatic palsy, cervical spine injury, or neuromuscular disease; (f) pneumothorax or pneumomediastinum; (g) use of muscle-paralyzing agents within 48 h before the study; (h) poor echocardiographic windows or difficult windows of diaphragmatic movement; (i) extubation failure definitely caused by upper airway obstruction; and (j) planned prophylactic noninvasive ventilation (NIV) after extubation.

### Study design

All patients were connected to a ventilator with pressure support ventilation [pressure support 10-12 cmH_2_O, positive end expiratory pressure 5 cmH_2_O, and proper fraction of inspired oxygen (FiO_2_) ≤ 40%] before the SBT. They fulfilled the SBT through a T-piece over 30 min in a supine position (30°- 45°). It took 5 to 15 min to perform TTE and diaphragm ultrasound before and after the SBT. The attending physicians remained blinded to the TTE and diaphragm ultrasound results. All patients should be extubated within 24 h once the SBT has succeeded. Medications, NIV or re-intubation could be performed in accordance with the patients’ conditions.

Both TTE and diaphragm ultrasound were performed using a Vivid i (GE Medical Systems Israel Ltd) with a 3.0 MHz US probe. TTE was done after diaphragm ultrasound. Diaphragmatic movement was measured by specific ultrasound doctors with the US probe placed over one of the lower intercostal spaces in the right anterior axillary line for the right diaphragm and left midaxillary line for the left diaphragm. The liver or spleen was used as a window for each hemidiaphragm. A two-dimensional mode was used to find the best approach and to select the exploration line for each hemidiaphragm. With the probe fixed on the chest wall during respiration, the ultrasound beam was directed to the hemidiaphragmatic domes at an angle of ≥70°; then, M-mode ultrasonography was used to display the motion of the anatomical structures along the selected line [11, 12, 14]. Unilateral diaphragmatic excursion (DE) equalled half of the sum of the values on the vertical axis of the tracing during the inspiration and expiration period, which were measured from baseline to the point of maximum height. DE (average) was equivalent to half of the sum of DE (right) and DE (left). Measurements were recorded at least six times and averaged for each hemidiaphragm. If the rhythm of respiration was not regular, measurements were needed at least ten times.

All patients were followed up with the events of respiratory failure within 48 h, and they were divided into respiratory failure and extubation success subgroups, accordingly. Respiratory failure was defined by at least two of the following criteria: arterial pH < 7.35 with arterial carbon dioxide tension (PaCO_2_) > 45 mmHg or > 20% higher than baseline; RR > 30 breaths/min or ≥ 50% higher than baseline; arterial oxygen tension (PaO_2_) < 60 mmHg or percutaneous oxygen saturation (SpO_2_) < 90% at FiO_2_ ≥ 0.5; decreased consciousness (e.g., coma, stupor, delirium), agitation, or diaphoresis; and clinical signs suggestive of respiratory muscle fatigue or increased work of breathing, such as retraction of intercostal spaces, use of accessory respiratory muscles, or thoracic-abdominal paradoxical movement [13, 15–17].

Then, each subgroup was assessed with respect to re-intubation within 1 week, and divided into re-intubation and non-intubation subgroups. The re-intubation should meet one of the following major criteria (a): respiratory or cardiac arrest, persistent severe hypoxemia (ratio of PaO_2_ to FiO_2_ ≤ 130 mmHg) despite NIV, haemodynamic instability with systolic blood pressure ≤ 85 mmHg despite adequate fluids and vasoactive drugs (dopamine or dobutamine ≥ 5 μg/kg/min or noradrenaline ≥ 0.05 μg/kg/min), and severe cardiac arrhythmia; or (b) at least two of the following minor criteria: clinical signs of severe acute respiratory failure with respiratory rate persistently > 35 breaths/min, arterial pH ≤ 7.20, worsening of acute respiratory failure under NIV, clinical signs suggestive of severely decreased consciousness (e.g., coma, stupor, and delirium) under NIV, bronchial hypersecretion under NIV, and development of other organ failure [13]. The attending doctor decided whether to re-intubate in the end. The re-intubation subgroup included the patients who were re-intubated or died within 1 week after extubation.

The criteria of readiness to wean and the SBT and NIV as well as, and the detailed methods for measuring E/Ea and left ventricular ejection fraction (LVEF) are described in Additional file 1.

Records were taken on the patients’ characteristics, including age, sex, body mass index (BMI), Acute Physiology and Chronic Health Evaluation (APACHE) II score at the time of admission to the ICU, comorbidities, duration from onset to the first intubation, duration of the first intubation to extubation, V_T_ and RSBI before the SBT, white blood cell count, haemoglobin, albumin, plasma creatinine and Glasgow coma scale (GCS) on the day of extubation. The data for DE, E, E/Ea and LVEF, arterial blood gas analysis, lactic acid and NT-pro-BNP were also collected.

### Statistical analysis

Statistical analysis was performed with SPSS 17.0 (Inc., Chicago, IL, USA). Data were expressed as the means ± SD or median quartile [first–third]. The Unpaired Student’s, Mann-Whitney, and the paired Wilcoxon tests were used to compare quantitative variables. Differences in the measurement data were compared with the one-way analysis of variance. Comparisons of proportions were performed with a Chi-square test or Fisher’s exact test. Risk factors predicting respiratory failure within 48 h or re-intubation within 1 week were selected by the backward-Wald method and analysed with a multivariate logistic regression model. The results were displayed in the last step. Meanwhile, the Hosmer-Lemeshow test was performed to verify the goodness of fit of the model. Then the area under the curve (AUC) and optimum cut-off value were identified by the receiver operating characteristic (ROC) curve. The level of significance was fixed at *P* ≤ 0.05.

## Results

**Fig. 1:**
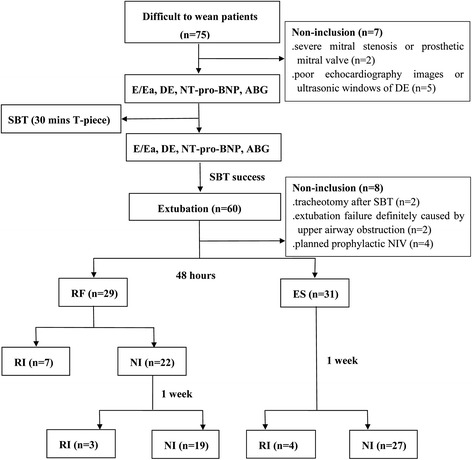
Flow chart. *ABG* arterial blood gas analysis, *DE* diaphragmatic excursion, *E/Ea* the ratio of mitral Doppler inflow velocity (E) to annular tissue Doppler wave velocity (Ea), *ES* extubation success, *NI* non-intubation, *NIV* noninvasive ventilation, *NT-pro-BNP* N-terminal-pro-B-type natriuretic peptide, *RF* respiratory failure, *RI* re-intubation, *SBT* spontaneous breathing trial

Figure 1 shows the flow-chart diagram of the study. Of the 75 patients who were screened, 60 patients (age range from 18 to 90 years -old) were enrolled. The reasons for intubation included severe pneumonia (*n* = 19), chronic obstructive airway disease (*n* = 10), cardiac events (*n* = 15), sepsis (*n* = 7), surgery (*n* = 8) and upper gastrointestinal haemorrhage (n = 1). The reasons for exclusion included severe mitral stenosis or prosthetic mitral valve (*n* = 2), poor echocardiography images or ultrasonic windows of diaphragm (*n* = 5), tracheotomy after SBT (*n* = 2), extubation failure caused by upper airway obstruction (*n* = 2) and planned prophylactic NIV (*n* = 4). Of 60 enrolled patients, 29 patients developed respiratory failure within 48 h after extubation, and 14 patients were re-intubated within 1 week.

### Predictors of respiratory failure within 48 h

**Fig. 2:**
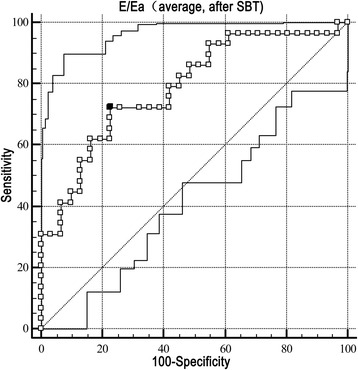
The ROC curve for E/Ea (average) after SBT predicting respiratory failure within 48 h. The AUC of E/Ea was 0.789 (*P* < 0.001). A cut-off value ≥ 12.5 of E/Ea (average) after SBT showed the highest diagnostic accuracy with a sensitivity and specificity of 72.4 and 77.4%, respectively. *AUC* area under curve, *E/Ea* the ratio of mitral Doppler inflow velocity (E) to annular tissue Doppler wave velocity (Ea), *ROC* receiver operating characteristic curve, *SBT* spontaneous breathing trial

**Fig. 3:**
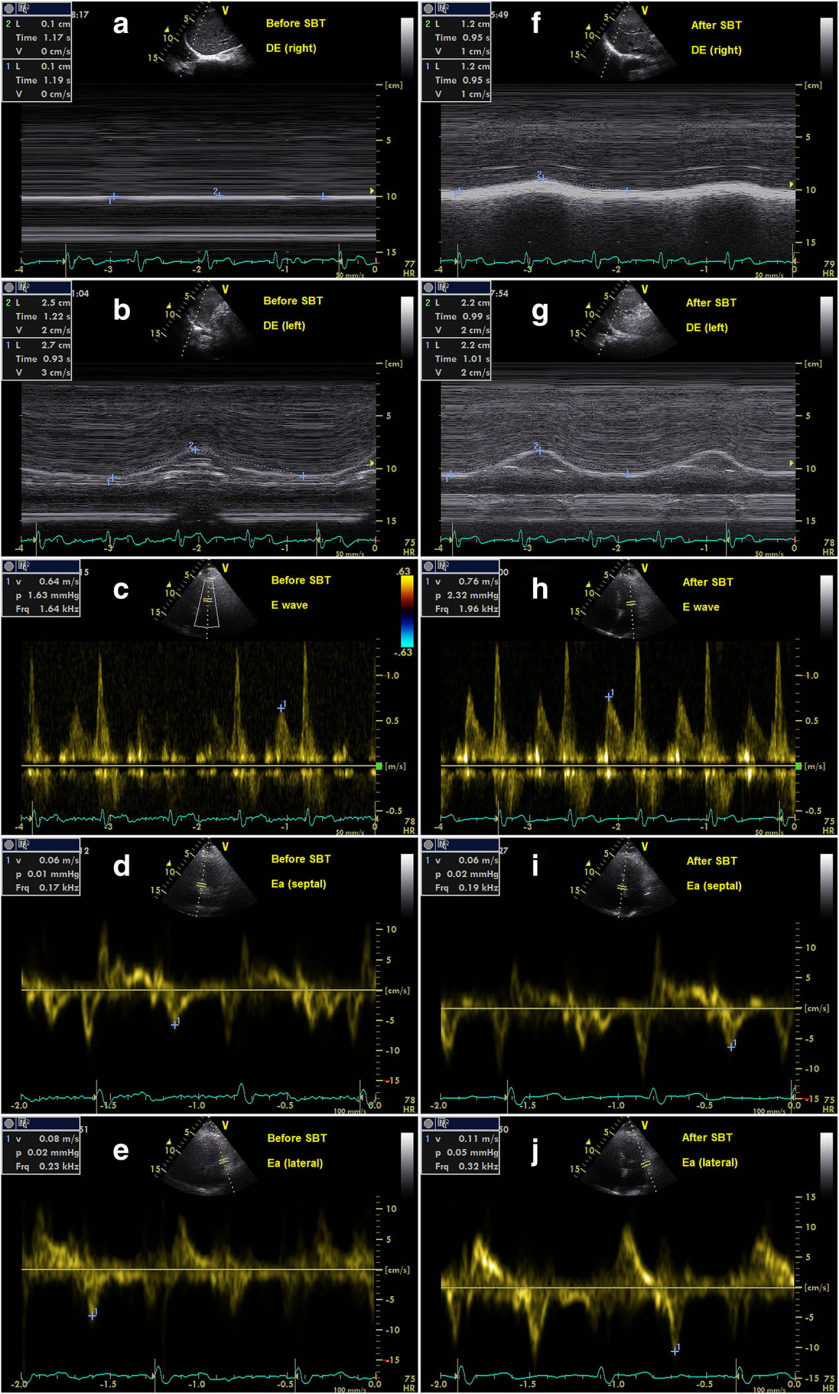
The measurements of E/Ea and DE before and after SBT in a patient of extubation success. **a**, **b**, **c**, **d**, **e** represented the results before SBT, and **f**, **g**, **h**, **i**, **j** represented the results after SBT. **a** DE (right) was 1 mm. **b** DE (left) was 26 mm. **c** E was 64 cm/s. **d** Ea (septal) was 6 cm/s. **e** Ea (lateral) was 8 cm/s. **f** DE (right) was 12 mm. **g** DE(left) was 22 mm. **h** E was 76 cm/s. **i** Ea (septal) was 6 cm/s. **j** Ea (lateral) was 11 cm/s. Before SBT, DE (average) was 13.5 mm, and E/Ea (average) was 9.1. After SBT, DE (average) was 17 mm, and E/Ea (average) was 8.9. After extubation, the patient did not develop respiratory failure within 48 h. The patient did not require re-intubation within 1 week. *DE* diaphragmatic excursion, *E/Ea* the ratio of mitral Doppler inflow velocity (E) to annular tissue Doppler wave velocity (Ea), *SBT* spontaneous breathing trial

**Fig. 4:**
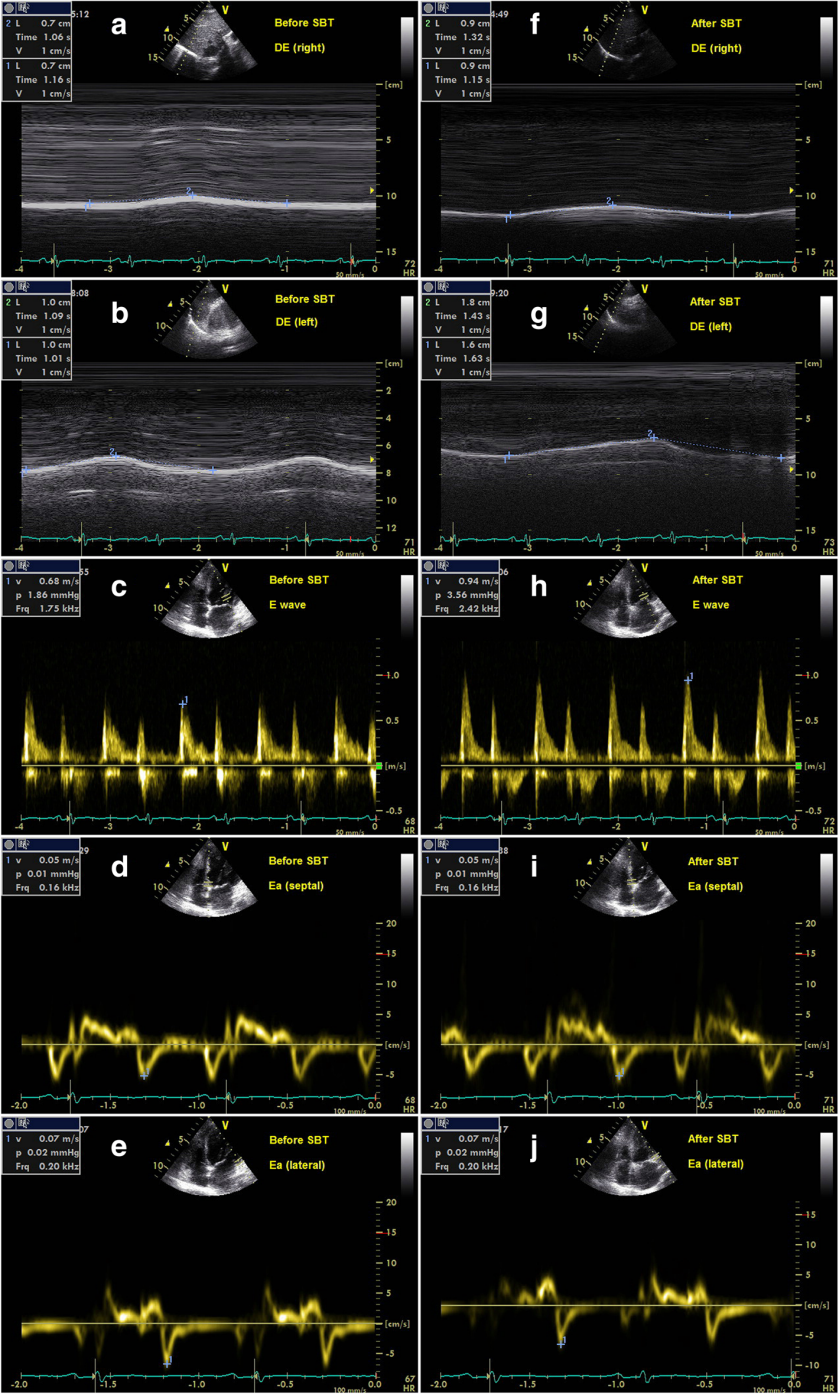
The measurements of E/Ea and DE before and after SBT in a patient of respiratory failure within 48 h. **a**, **b**, **c**, **d**, **e** represented the results before SBT, and **f**, **g**, **h**, **i**, **j** represented the results after SBT. **a** DE (right) was 7 mm. **b** DE (left) was 10 mm. **c** E was 68 cm/s. **d** Ea (septal) was 5 cm/s. **e** Ea (lateral) was 7 cm/s. **f** DE (right) was 9 mm. **g** DE (left) was 17 mm. **h** E was 94 cm/s. **i** Ea (septal) was 5 cm/s. **j** Ea (lateral) was 7 cm/s. Before SBT, DE (average) was 8.5 mm, and E/Ea (average) was 11.3. After SBT, DE (average) was 13 mm, and E/Ea (average) was 15.7. After extubation, respiratory failure occurred within 48 h and then NIV was used. The patient was not re-intubated within 1 week and free of NIV in the end. *DE* diaphragmatic excursion, *E/Ea* the ratio of mitral Doppler inflow velocity (E) to annular tissue Doppler wave velocity (Ea), *NIV* noninvasive ventilation, *SBT* spontaneous breathing trial

Among 60 patients, 29 cases (48%) developed respiratory failure within 48 h after extubation (respiratory failure subgroup, RF subgroup), and another 31 cases (52%) were successfully extubated (extubation success subgroup, ES subgroup). There were significant differences in some clinical characteristics between the RF and ES subgroups (Table 1). Partial differences were also seen in the laboratory and ultrasound data (Table 2). Multivariate logistic regression showed that E/Ea (average) after SBT [odds ratio (OR) 1.450, 95% confidence intervals (CI) 1.092-1.926, *P* = 0.01] and the LVEF (OR 0.904, CI 0.833-0.981, *P* = 0.015) were closely related to respiratory failure within 48 h. The *P* value for the Hosmer-Lemeshow test was 0.11. The AUC of E/Ea (average) after SBT was 0.789, and a cut-off value ≥12.5 showed the highest diagnostic accuracy with a sensitivity and specificity of 72.4% and 77.4%, respectively (Fig. 2). The results of logistic regression and the ROC curve in this study were summarized in Table 3. For example, compared with a case of extubation success (Fig. 3), E/Ea (average) after SBT was high in a patient in the RF subgroup (Fig. 4).

**Table 1:** Clinical characteristics

	Respiratory failure within 48 h	
Variables	RF subgroup	ES subgroup
N (cases)	29	31
Demographic factors:		
Age (yrs)	70.8 ± 15.6	62.3 ± 22.2
Male	18 (62)	17 (55)
BMI (kg/m^2^)	24.4 ± 5.3	24.3 ± 4.3
Comorbidities:		
Chronic respiratory diseases^a^	19 (66)	7 (23)*
Coronary heart disease	16 (55)	7 (23)*
Myocardial infarction	11 (38)	6 (19)
Hypertension	17 (59)	19 (61)
Atrial fibrillation	8 (28)	3 (32)
Diabetes	11 (38)	12 (39)
Renal dysfunction	10 (29)	5 (16)
Cerebral vascular diseases^b^	6 (21)	4 (13)
Clinical characteristics:		
APACHE II	23.9 ± 4.7	20 ± 6.4*
Duration from onset to the first intubation (d)	6.3 [1.5–9]	3.6 [1–6]*
Duration of the first intubation to extubation (d)	10.4 ± 6.9	7.2 ± 4.7*
RR (breaths/min)	21.6 ± 4.3	21.5 ± 4.7
V_T_ (l)	0.43 ± 0.12	0.41 ± 0.09
RSBI	55.8 ± 22.2	55.9 ± 22.1
GCS	13.4 [13–15]	13.8 [13–15]
WBC (×10^9^/l)	9.8 ± 3.9	10.9 ± 5.6
Hemoglobin (g/l)	103 ± 23	89 ± 14*
Albumin (g/l)	28.8 ± 4.2	27.7 ± 3.9
Plasma creatinine (micromole/l)	121 ± 74	115 ± 80

Data are presented as median ± SD or median (IQR) or n (%). *APACHE II* Acute Physiology and Chronic Health Evaluation II, *BMI* body mass index, *ES* extubation success, *GCS* Glasgow coma scale, *RF* respiratory failure, *RR* respiratory rate, *RSBI* rapid shallow breathing index, *V*_*T*_ tidal volume, *WBC* white blood cell. ^a^Asthma, obsolete tuberculosis, chronic obstructive pulmonary disease, bronchiectasis and interstitial lung disease are included. ^b^Cerebral infarction, cerebral hemorrhage and subarachnoid hemorrhage are included. * Significant at *p* < 0.05

**Table 2:** Laboratory and ultrasonic data before and after SBT

	Respiratory failure within 48 h	
Variables	RF subgroup	ES subgroup
N (cases)	29	31
Before SBT		
PH	7.45 ± 0.06	7.46 ± 0.05
PaCO_2_ (mmHg)	46.4 ± 13	39.1 ± 5.1*
PaO_2_/FiO_2_	277 ± 83	281 ± 80
Lactic acid (mmol/l)	1.2 ± 0.6	1.1 ± 0.4
NT-pro-BNP(pg/ml)	6519 ± 9162	1578 ± 1843*
Pleural effusion	22 (76)	24 (77)
LVEF (%)	57 ± 14	64 ± 9*
E (cm/s)	92.9 ± 25.6	78.2 ± 18.4*
E/Ea (septal)	18.2 ± 8.1	13 ± 3.8*
E/Ea (lateral)	12.8 ± 5.1	8.4 ± 2.7^†^
E/Ea (average)	14.7 ± 5.6	10.1 ± 2.8^†^
DE (right) (mm)	11.2 ± 4.7	10.1 ± 4.7
DE (left) (mm)	11.6 ± 6.3	10.8 ± 5.5
DE (average) (mm)	11.4 ± 4.5	10.5 ± 3.4
After SBT		
PH	7.43 ± 0.05	7.47 ± 0.05*
PaCO_2_ (mmHg)	48 ± 11.7	38.7 ± 5.5^†^
PaO_2_/FiO_2_	291 ± 103	275 ± 79
Lactic acid (mmol/l)	1.1 ± 0.5	1.1 ± 0.5
NT-pro-BNP(pg/ml)	6518 [421-10,477]	1471[176-1575]*
E (cm/s)	99.7 ± 24.3	85.1 ± 18.2*
E/Ea (septal)	18.6 ± 6.5	13.4 ± 4.4*
E/Ea (lateral)	14.4 ± 7.3	9.0 ± 3.0*
E/Ea (average)	15.8 ± 6.2	10.6 ± 3.2^†^
DE (right) (mm)	13.7 ± 6	11 ± 4.4
DE (left) (mm)	13.8 ± 6.4	13 ± 5.4
DE (average) (mm)	13.7 ± 5.4	12 ± 3.4

Data are presented as median ± SD or median (IQR) or n (%). *DE* diaphragmatic excursion, *E* mitral Doppler early peak diastolic velocity, *E/Ea* the ratio of mitral Doppler inflow velocity to annular tissue Doppler wave velocity, *ES* extubation success, *LVEF* left ventricular ejection fraction, *NT-pro-BNP* N-terminal-pro-B-type natriuretic peptide, *PaCO*_*2*_ arterial carbon dioxide tension, *PaO*_*2*_ */FiO*_*2*_ the ratio of arterial oxygen tension to fraction of inspired oxygen, *RF* respiratory failure, *SBT* spontaneous breathing trial. * Significant at *p* < 0.05, and ^†^ significant at *p* < 0.001

**Table 3:** The parameters of the ROC curve of the predictors of respiratory failure within 48 h

Variables	OR	95% CI	*P-*value	Cut-off value	AUC	Sensitivity	Specificity
Chronic respiratory diseases	5.013	0.738–34.067	0.099	NA	NA	NA	NA
Duration from onset to the first intubation	1.269	0.989–1.627	0.061	NA	NA	NA	NA
PH after SBT	< 0.001	0–3.322	0.066	NA	NA	NA	NA
PaCO_2_ after SBT	1.146	0.980–1.340	0.088	NA	NA	NA	NA
LVEF	0.904	0.833–0.981	0.015	≤ 52	0.646	34.5%	96.8%
E/Ea (average) after SBT	1.450	1.092–1.926	0.010	≥ 12.5	0.789	72.4%	77.4%

*AUC* area under curve, *CI* confidence interval, *E/Ea* the ratio of mitral Doppler inflow velocity to annular tissue Doppler wave velocity, *LVEF* left ventricular ejection fraction, *NA* not available, *PaCO*_*2*_ arterial carbon dioxide tension, *OR* odds ratio, *ROC* receiver operating characteristic curve, *SBT* spontaneous breathing trial

Meanwhile, NT-pro-BNP was analysed separately from the E/Ea and LVEF because of their significant correlations. Multivariate logistic regression analysis showed that the NT-pro-BNP level and PaCO_2_ after SBT, duration of the first intubation to extubation and haemoglobin were closely related to respiratory failure within 48 h (Additional file 1: Table S1). However, the OR value of NT-pro-BNP was very close to 1.

### Predictor of re-intubation within 1 week in the respiratory failure subgroup

**Fig. 5:**
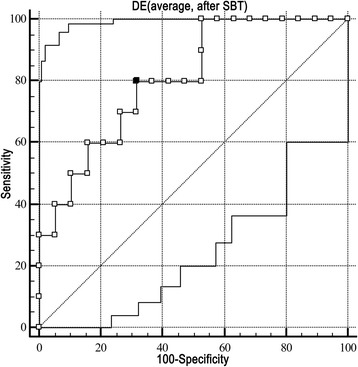
The ROC curve for DE (average) after SBT predicting re-intubation within 1 week in the respiratory failure subgroup. The AUC of DE (average) after SBT was 0.805 (*P* = 0.008). A cut-off value ≤ 12.6 mm showed the highest diagnostic accuracy with a sensitivity and specificity of 80% and 68.4%, respectively. *AUC* area under curve, *DE* diaphragmatic excursion, *ROC* receiver operating characteristic curve, *SBT* spontaneous breathing trial

Among all patients, 29 cases developed postextubation respiratory failure within 48 h. These 29 cases received NIV, and 4 of them experienced re-intubation within 48 h. However, 6 of the remaining of 25 cases required re-intubation within 1 week. Of 10 cases with re-intubation, 6 patients were re-intubated for heart failure and 4 patients for ineffective cough. One-way analysis of variance showed significant differences for DE (right) and DE (average) after SBT, the haemoglobin and BMI (Tables 4 and 5). However, multivariate logistic regression analysis showed that only DE (average) after SBT was associated with re-intubation within 1 week (OR 0.690, CI 0.499–0.953, *P* = 0.024) (Table 6). The *P* value for the Hosmer-Lemeshow test was 0.559. The AUC of DE (average) after SBT was 0.805, and the cut-off value ≤ 12.6 mm showed the highest diagnostic accuracy with a sensitivity and specificity of 80% and 68.4%, respectively (Fig. 5).

**Table 4:** Clinical characteristics in the respiratory failure and extubation success subgroups

	Respiratory failure subgroup		Extubation success subgroup	
Variables	RI subgroup	NI subgroup	RI subgroup	NI subgroup
N (cases)	10	19	4	27
Demographic factors:				
Age (yrs)	70.1 ± 21.5	71.2 ± 12.2	70.0 ± 11.5	61.2 ± 23.3
Male	6 (60)	12 (63)	4 (100)	13 (48)
BMI (kg/m^2^)	21.6 ± 4.1	25.9 ± 5.4*	23.9 ± 1.1	24.4 ± 4.5
Comorbidities:				
Chronic respiratory diseases^a^	7 (70)	12 (63)	1 (25)	6 (22)
Coronary heart disease	5 (50)	11 (58)	1 (25)	6 (22)
Myocardial infarction	2 (20)	9 (47)	1 (25)	5 (19)
Hypertension	5 (50)	12 (63)	3 (75)	16 (59)
Atrial fibrillation	4 (40)	4 (21)	0 (0)	3 (11)
Diabetes	2 (20)	6 (32)	2 (50)	8 (30)
Renal dysfunction	6 (60)	5 (26)	3 (75)	9 (33)
Cerebral vascular diseases^b^	4 (40)	2 (11)	1 (25)	3 (11)
Clinical characteristics:				
APACHE II	26.2 ± 6.4	22.7 ± 3.0	24.0 ± 5.0	19.3 ± 6.4
Duration from onset to the first intubation (d)	9.0 ± 7.0	5.0 ± 3.4	1.0 ± 0.8	4.0 ± 4.0*
Duration of the first intubation to extubation (d)	13.6 ± 9.6	8.7 ± 4.4	5.8 ± 3.0	7.4 ± 4.9
RR (breaths/min)	22.5 ± 5.6	21.2 ± 3.5	23 ± 6.7	21.3 ± 4.4
V_T_ (l)	0.40 ± 0.12	0.44 ± 0.12	0.38 ± 0.09	0.41 ± 0.09
RSBI	63.9 ± 33.1	51.6 ± 12.8	63.9 ± 29.0	54.8 ± 21.3
GCS	12.4 ± 3.6	14.0 ± 1.0	13.5 [13-14]	13.9 [13-15]
WBC (×10^9^/l)	8.5 ± 4.1	10.6 ± 3.6	8.8 ± 3.7	11.3 ± 5.8
Hemoglobin (g/l)	87.2 ± 12.0	111.5 ± 23.3*	81.0 ± 16.8	90.6 ± 14.0
Albumin (g/l)	28.9 ± 5.1	28.8 ± 3.9	25.3 ± 3.3	28.0 ± 3.9
Plasma creatinine (micromole/l)	134.9 ± 83.0	113.0 ± 70.7	155.4 ± 90.5	109.4 ± 79.0

Data are in median ± SD or median (IQR) or n (%). *APACHE II* Acute Physiology and Chronic Health Evaluation II, *BMI* body mass index, *GCS* Glasgow coma scale, *NI* non-intubation, *RI* re-intubation, *RR* respiratory rate, *RSBI* rapid shallow breathing index, *V*_*T*_ tidal volume, *WBC* white blood cell. ^a^Asthma, obsolete tuberculosis, chronic obstructive pulmonary disease, bronchiectasis and interstitial lung disease. ^b^Cerebral infarction, cerebral hemorrhage and subarachnoid hemorrhage. *p < 0.05

**Table 5:** Laboratory and ultrasonic data in the respiratory failure and extubation success subgroups before and after SBT

	Respiratory failure subgroup		Extubation success subgroup	
Variables	RI subgroup	NI subgroup	RI subgroup	NI subgroup
N (cases)	10	19	4	27
Before SBT				
PH	7.44 ± 0.06	7.46 ± 0.06	7.47 ± 0.05	7.46 ± 0.05
PaCO_2_ (mmHg)	51.1 ± 15.3	44.2 ± 11.6	38.8 ± 9.5	39.1 ± 4.4
PaO_2_/FiO_2_	311 ± 88	261 ± 77	304 ± 99	278 ± 78
Lactic acid (mmol/l)	1.2 ± 0.6	1.1 ± 0.7	1.1 ± 0.8	1.1 ± 0.3
NT-pro-BNP (pg/ml)	8907 ± 10,180	5262 ± 8597	3043 ± 2014	1344 ± 1744
Pleural effusion	7 (70)	15 (79)	4 (100)	20 (74)
LVEF (%)	58 ± 16	57 ± 14	58.5 ± 7.9	65.3 ± 8.9
E (cm/s)	97.0 ± 24.6	90.7 ± 26.4	82.1 ± 28.6	77.6 ± 17.2
E/Ea (septal)	17.2 ± 11.0	18.7 ± 6.4	12.8 ± 5.9	13.0 ± 3.6
E/Ea (lateral)	11.7 ± 6.0	13.4 ± 4.6	6.6 ± 3.0	8.7 ± 2.6
E/Ea (average)	13.5 ± 6.7	15.4 ± 5.1	8.6 ± 3.9	10.3 ± 2.6
DE (right) (mm)	9.6 ± 4.4	12.1 ± 4.8	6.0 ± 1.4	10.7 ± 4.7
DE (left) (mm)	10.4 ± 5.1	12.3 ± 6.9	8.3 ± 3.7	11.2 ± 5.6
DE (average) (mm)	10.0 ± 3.4	12.2 ± 4.9	7.2 ± 2.3	11.0 ± 3.2*
After SBT				
PH	7.43 ± 0.05	7.44 ± 0.06	7.42 ± 0.05	7.47 ± 0.04*
PaCO_2_ (mmHg)	51.5 ± 14.2	46.1 ± 10.1	39.3 ± 7.0	38.6 ± 5.4
PaO_2_/FiO_2_	317 ± 107	278 ± 101	307 ± 59	271 ± 81
Lactic acid (mmol/l)	1.1 ± 0.5	1.1 ± 0.6	1.5 ± 1.0	1.0 ± 0.3
NT-pro-BNP (pg/ml)	8778 ± 11,082	5329 ± 8654	3631 ± 3273	1138 ± 1517*
E (cm/s)	99.2 ± 27.3	99.9 ± 23.3	83.5 ± 23.5	85.3 ± 17.8
E/Ea (septal)	17.8 ± 7.8	19.0 ± 5.8	12.6 ± 5.3	13.5 ± 4.4
E/Ea (lateral)	13.2 ± 8.0	15.0 ± 7.0	7.2 ± 2.5	9.2 ± 3.0
E/Ea (average)	14.6 ± 7.0	16.4 ± 5.9	9.1 ± 3.4	10.8 ± 3.1
DE (right) (mm)	8.9 ± 4.6	16.2 ± 5.1*	8.2 ± 2.4	11.5 ± 4.5
DE (left) (mm)	10.7 ± 5.3	15.5 ± 6.4	9.6 ± 2.4	13.5 ± 5.5
DE (average) (mm)	9.8 ± 3.6	15.8 ± 5.1*	8.9 ± 1.9	12.5 ± 3.4*

Data are in median ± SD or median (IQR) or n (%). *DE* diaphragmatic excursion, *E* mitral Doppler early peak diastolic velocity, *E/Ea* the ratio of mitral Doppler inflow velocity to annular tissue Doppler wave velocity, *LVEF* left ventricular ejection fraction, *NI* non-intubation, *NT-pro-BNP* N-terminal-pro-B-type natriuretic peptide, *PaCO*_*2*_ arterial carbon dioxide tension, *PaO*_*2*_ */FiO*_*2*_ the ratio of arterial oxygen tension to fraction of inspired oxygen, *RI* re-intubation, *SBT* spontaneous breathing trial. *p < 0.05

**Table 6:** The parameters of the ROC curve of the predictor of re-intubation within 1 week in the respiratory failure subgroup

Variables	OR	95% CI	*P-*value	Cut-off value	AUC	Sensitivity	Specificity
DE (average) after SBT	0.690	0.499-0.953	0.024	≤ 12.6	0.805	80%	68.4%

*AUC* area under curve, *CI* confidence interval, *DE* diaphragmatic excursion, *OR* odds ratio, *ROC* receiver operating characteristic curve, *SBT* spontaneous breathing trial

### Risk factors for re-intubation within 1 week in the extubation success subgroup

Among 31 patients in the extubation success subgroup, 4 cases were re-intubated within 1 week after extubation (2 cases for abdominal infection, 1 case for ineffective cough and 1 case for heart failure). One-way analysis of variance showed significant differences for the duration from onset to the first intubation, PH after SBT, NT-pro-BNP after SBT, and DE (average) before and after SBT (Tables 4 and 5). However, multivariate logistic regression analysis showed none of these factors were associated with re-intubation within 1 week (Additional file 1: Table S2).

## Discussion

The most important findings in this study confirmed the different effects of cardiac and diaphragm function assessed by ultrasound on extubation outcomes in difficult-to-wean patients. Our results indicated that E/Ea (average) after SBT could predict respiratory failure within 48 h, and DE (average) after SBT might predict re-intubation within 1 week in the respiratory failure subgroup. These results supported that cardiac diastolic function occurred in the short term after extubation, and diaphragm dysfunction persisted in the long term after extubation.

On the one hand, during abrupt transfer from mechanical ventilation to spontaneous breathing, decreases in intrathoracic pressures would augment venous return and intrathoracic blood volume, increasing the cardiac preload. Furthermore, increased left ventricular transmural pressures would augment cardiac afterload during the process. Otherwise, the increase in the breathing work and discharge of catecholamine might increase the cardiac work and myocardial oxygen demand [18]. Therefore, cardiac diastolic function would become worse and could lead to postextubation respiratory failure quickly. On the other hand, diaphragm dysfunction usually occurred with chronic respiratory diseases or abdominal diseases, which were often accompanied with refractory respiratory failure or ineffective cough. These were two important reasons for re-intubation. Therefore, diaphragm dysfunction could be associated with re-intubation. Furthermore, other accessory respiratory muscles or NIV could partially compensate for diaphragm dysfunction. As a result, re-intubation often occurred in the long term, compared to postextubation respiratory failure.

In this study, we demonstrated that E/Ea (average) after SBT could predict respiratory failure within 48 h, and that a cut-off value ≥12.5 had the highest predictive value. These conclusions were partially in line with the results of early case studies [6–9]. However, three issues should be addressed. First, E/Ea (average) but not E/Ea (septal or lateral) after SBT was identified as an ideal predictor in this study; because 17 cases (28%) were diagnosed with myocardial infarction in this study. E/Ea (average) instead of E/Ea (septal or lateral) was recommended to reflect cardiac diastolic function in the presence of regional dysfunction by the Guidelines [19]. Second, the NT-pro-BNP level was higher in the respiratory failure subgroup, but further analysis showed that the OR value was very close to 1, which limited its predictive value. As the NT-pro-BNP was easily affected by multiple factors, it might be more suitable for E/Ea (average) to accurately reflect cardiac diastolic dysfunction related to extubation. Finally, there were no differences in DE between the respiratory failure and extubation success subgroups, indicating that diaphragm dysfunction did not play an important role in respiratory failure within 48 h. Other accessary respiratory muscles could partially compensate for diaphragm dysfunction. Therefore, it often took a long time for diaphragm dysfunction to cause respiratory failure, compared to cardiac dysfunction. A study by Carrie et al. showed that DE could predict weaning failure with a low sensitivity and specificity of 59% and 71%, respectively [20]. Weaning failure was defined as SBT failure, respiratory failure or death within 48 h after extubation. Although there were partial differences between the criteria for weaning failure and those for postextubation respiratory failure, DE seemed not to be significantly associated with respiratory failure within 48 h.

In the respiratory failure subgroup, DE (average) after SBT was the only protective factor associated with re-intubation within 1 week, which was a significant finding. The cut-off value ≤12.6 mm showed the highest diagnostic accuracy. As the diaphragm serves as a main part in the formation of the cough reflex arc, its function can partly reflect the ability to cough and discharge sputum. Many studies have indicated that cough strength is a good predictor of extubation failure [21–24]. Diaphragm dysfunction was closely related to ineffective cough and primary respiratory failure, which were considered two important reasons for re-intubation. Therefore, diaphragm dysfunction could help predict re-intubation. Jiang JR et al. reported that the movements of the liver and spleen during SBT could predict extubation success within 72 h, and that the cut-off value of 1.1 cm had the greatest sensitivity (84.4%) and specificity (82.6%) [10]. Kim WY et al. demonstrated that patients with diaphragm dysfunction, defined as DE < 10 mm or paradoxical movements during SBT, had higher rates of primary weaning failure and secondary weaning failure [11]. Our conclusions were partially consistent with these studies. However, three problems should be highlighted. First, DE (average) after SBT instead of DE (right) after SBT was identified as an ideal predictor in this study. In fact, the discrepancy of bilateral diaphragmatic movement was very common in this study, which was similar to another study mentioned above [11]. Unilateral diaphragm dysfunction could be compensated by the reverse side. Therefore, DE (average) could more accurately reflect the overall diaphragm function. Second, all patients received NIV in this subgroup, but 10 cases (34%) required re-intubation within 1 week. Currently, NIV as an early weaning technique from mechanical ventilation is controversial [13]. NIV can be used to partially improve respiratory muscular fatigue and cardiac dysfunction, but it remains a challenge to correct ineffective cough. Diaphragm function can partially reflect the ability of cough. Therefore, diaphragm ultrasound could partially help identify patients with a high risk for re-intubation. Finally, Spadaro S et al. demonstrated that the diaphragmatic-rapid shallow breathing index (D-RSBI) could predict weaning failure with a high sensitivity of 94.1% and a specificity of 64.7%, and the AUC was 0.89 [25]. D-RSBI was a good comprehensive index that could reflect both the diaphragm function and presence of rapid shallow breathing, a sign of overall imbalance between the mechanical load posed on the diaphragm and its ability to face the load. On the other hand, D-RSBI could easily be affected by multiple factors such as the ability of the accessory respiratory muscles, the impaired diaphragmatic function, weaning-induced cardiac failure, excess mechanical load, and more. Therefore, D-RSBI was a good predictor of weaning failure. However, we analysed the data of D-RSBI in this study and found that there were no significant differences between the re-intubation and non-intubation subgroups (Additional file 1: Table S3). Compared to D-RSBI, DE was a simple and direct index reflecting diaphragm function. DE could partially reflect the severity of primary respiratory failure and ineffective cough, which were considered two important reasons for re-intubation. Therefore, DE could be a better predictor of re-intubation than D-RSBI.

Furthermore, one-way analysis of variance showed that DE (average) after SBT was a protective factor for re-intubation within 1 week in the extubation success subgroup. However, further analysis showed that there were no correlations. These results could be explained by the small sample size and poorly balanced distribution in the re-intubation and non-intubation subgroups (4 and 27 cases).

In summary, the highlight of this study was that E/Ea (average) after SBT could predict respiratory failure within 48 h, and DE (average) after SBT might predict re-intubation within 1 week in the respiratory failure subgroup. This was an interesting phenomenon, which implied that cardiac diastolic dysfunction occurred quickly after extubation and could be partially improved by NIV and medication. On the other hand, diaphragm dysfunction often persisted in the long term after extubation. It was difficult to correct the dysfunction quickly even by NIV and medications.

It was noteworthy that severe diaphragm dysfunction could also lead to postextubation respiratory failure within 48 h. In the study by Kim WY et al., DE medians were 7.0 mm and 7.9 mm in the right and left diaphragm in the diaphragmatic dysfunction group, respectively, which could explain a high incidence (83%) of weaning failure within 48 h in this group [11]. Another study by Spadaro S et al. showed that DE could predict weaning failure, with an AUC of 0.82 [25]. The average value of DE in the right diaphragm was 7.0 mm in the weaning failure group, which was remarkably lower than 13.7 mm in the respiratory failure subgroup in our study. Therefore, the lower the value of DE, the earlier respiratory failure might occur after extubation. On the other hand, it is generally known that severe cardiac dysfunction can also cause re-intubation even by NIV and medication. Therefore, clinicians should analyse specific case features and assess the cardiac and diaphragm function together using bedside ultrasound in the weaning process.

### Study limitations

This study has several limitations. First, different proportions of indications for intubation could have resulted in different conclusions. Therefore, it was helpful to analyse the results in different subgroups. However, 60 cases had a well-balanced distribution for the main reasons for intubation such as chronic respiratory diseases (*n* = 10), severe pneumonia (*n* = 19) and cardiac diseases (*n* = 15). Therefore, the predictive value of E/Ea and DE can be evaluated in different diseases, such as chronic obstructive pulmonary disease or acute myocardial infarction, in the future. Second, the sample size in this study was small. Although DE (average) after SBT was one of protective factors for re-intubation within 1 week in the extubation success subgroup, further analysis showed that there were no correlations. Therefore, it is necessary to enroll sufficient patients to validate these conclusions in both respiratory failure and extubation success subgroups. Third, the percent change in the diaphragm thickness between end-expiration and end-inspiration was identified as a good predictor of extubation success [12, 26, 27]. However, we did not take this indicator into consideration at the beginning in this study. We are planning on adding the percent change in diaphragm thickness to analysis in our next study. Fourth, neither E/Ea nor DE can reflect other aetiologies, such as excess secretions, upper airway obstruction, mental status, etc. Therefore, extubation outcomes should continue to be based on specific case features. Finally, the GCS score, instead of the Richmond Agitation and Sedation Scale (RASS) score, was used to evaluate the level of sedation before SBT. The RASS score is more accurate and appropriate than the GCS score. Although there are no significant differences in the GCS score between different subgroups in this study, it is necessary for the RASS score to be used to assess the effect of extubation outcomes in a future study.

## Conclusions

E/Ea (average) after SBT could help predict respiratory failure within 48 h after extubation. However, DE (average) after SBT could help predict re-intubation within 1 week in the respiratory failure subgroup. TTE and diaphragm ultrasound should be performed in different diseases to confirm these conclusions.

## Additional file

Additional file 1: The criteria for readiness to wean and SBT and NIV, and the detailed methods for measuring E/Ea and LVEF. **Table S1.** The parameters of the predictors of respiratory failure within 48 h including NT-pro-BNP (after SBT). **Table S2.** The parameters of the predictors of re-intubation within 1 week in the extubation success subgroup. **Table S3.** The D-RSBI in the re-intubation and non-intubation subgroups. (DOC 67 kb)

## Abbreviations

APACHE: Acute physiology and chronic health evaluation
AUC: Area under curve
BNP: B-type natriuretic peptide
CI: Confidence interval
DE: Diaphragmatic excursion
D-RSBI: Diaphragmatic-rapid shallow breathing index
E/A: The ratio of mitral Doppler early peak diastolic velocity to late peak diastolic velocity
E/Ea: The ratio of mitral Doppler inflow velocity (E) to annular tissue Doppler wave velocity (Ea)
ES subgroup: Extubation success subgroup
FiO_2_: Proper fraction of inspired oxygen
GCS: Glasgow coma scale
ICU: Intensive care units
LVEF: Left ventricular ejection fraction
NI subgroup: Non-intubation subgroup
NIV: Noninvasive ventilation
NT-pro-BNP: N-terminal-pro-BNP
OR: Odds ratio
PaCO_2_: Arterial carbon dioxide tension
PaO_2_: Arterial oxygen tension
RASS: Richmond agitation and sedation scale
RF subgroup: Respiratory failure subgroup
RI subgroup: Re-intubation subgroup
ROC: Receiver operating characteristic
RR: Respiratory rate
RSBI: Rapid shallow breathing index
SBT: Spontaneous breathing trial
SpO_2_: Percutaneous oxygen saturation
TTE: Transthoracic echocardiography
V_T_: Tidal volume
