# Supplementary material for: Different effects of cardiac and diaphragm function assessed by ultrasound on extubation outcomes in difficult-to-wean patients: a cohort study
Source: BMC Pulm Med. 2017 Dec 1;17:161. doi: 10.1186/s12890-017-0501-8 (PMC5709823; doi:10.1186/s12890-017-0501-8)
Supplement: Additional file 1: — The criteria for readiness to wean and SBT and NIV, and the detailed methods for measuring E/Ea and LVEF. Table S1. The parameters of the predictors of respiratory failure within 48 h including NT-pro-BNP (after SBT). Table S2. The parameters of the predictors of re-intubation within 1 week in the extubation success subgroup. Table S3. The D-RSBI in the re-intubation and non-intubation subgroups. (DOC 67 kb) [file 12890_2017_501_MOESM1_ESM.doc]

**Different effects of cardiac and diaphragm function assessed by ultrasound on extubation outcomes in difficult-to-wean patients: a cohort study**

**Criteria**

All patients were connected to ventilator with pressure support ventilation before the spontaneous breathing trial (SBT). The criteria for readiness to wean: adequate cough, absence of excessive tracheobronchial secretion, resolution of disease acute phase for which the patient was intubated, stable cardiovascular status [heart rate (HR) ≤ 140 beats/min, systolic arterial pressure (SBP) 90 - 160 mmHg with no or minimal vasopressors], stable metabolic status, adequate oxygenation [percutaneous oxygen saturation (SpO_2_) > 90% on proper fraction of inspired oxygen (FiO_2_) ≤ 40% with positive end expiratory pressure (PEEP) ≤ 8 cmH_2_O], adequate mentation .

A SBT was performed over a 30 minute period using a T-piece while the patients lay in a supine position (30°-45°) with FiO_2_ ≤ 50%. SBT failure was defined at least one of the following reasons: agitation and anxiety, depressed mental status, cyanosis, arterial pH < 7.32, arterial carbon dioxide tension (PaCO_2_) > 10 mmHg than baseline, arterial oxygen tension (PaO_2_) ≤ 60 mmHg at FiO_2_ ≥ 0.5, respiratory rate (RR) ≥ 35 breathes/min, HR > 140 beats/min or cardiac arrhythmia, SBP > 180 mmHg or < 90 mmHg . If a SBT was successful, the planned extubation was performed in the next 24 hours.

Noninvasive ventilation (NIV) could be applied in the case of postextubation respiratory failure. The attending doctor decided whether to use NIV according to patients' conditions, consciousness and [tolerance](../../../../C:/Users/lenovo/Desktop/chest/罗凌-chest/../../AppData/Local/Yodao/DeskDict/frame/20150728214337/javascript:void(0)%3B) in the end.

**Methods**

The patients lay in a supine position (30°- 45°). The left ventricular ejection fraction (LVEF) was assessed using biplane Simpson’s method from the apical two- and four-chamber views . Pulsed-wave Doppler analysis of mitral inflow allowed the measurement of the early peak diastolic velocity (E) at the tip of the mitral valve leaflets. The myocardial velocity was recorded using the pulse-wave tissue Doppler, which was positioned at the septal and lateral mitral annulus in apical four-chamber view, and early diastolic velocities (Ea) were measured. Then E/Ea (septal) and E/Ea (lateral) were calculated. Ea (average) equaled half of the sum of the values of Ea (septal) and Ea (lateral), so E/ Ea (average) could be also calculated according to the guidelines . At least five measurements were used to obtain the average values. In the case of atrial fibrillation, frequent atrial or ventricular premature beats, ventricular or supraventricular tachycardia, E/Ea was averaged over 10 cardiac cycles .

**References**

**1. Boles JM, Bion J, Connors A, Herridge M, Marsh B, Melot C, Pearl R, Silverman H, Stanchina M, Vieillard-Baron A *et al*: Weaning from mechanical ventilation. *Eur Respir J* 2007, 29(5):1033-1056.**

**2. MacIntyre NR, Cook DJ, Ely EW, Jr., Epstein SK, Fink JB, Heffner JE, Hess D, Hubmayer RD, Scheinhorn DJ: Evidence-based guidelines for weaning and discontinuing ventilatory support: a collective task force facilitated by the American College of Chest Physicians; the American Association for Respiratory Care; and the American College of Critical Care Medicine. *Chest* 2001, 120(6 Suppl):375S-395S.**

**3. Schiller NB, Shah PM, Crawford M, DeMaria A, Devereux R, Feigenbaum H, Gutgesell H, Reichek N, Sahn D, Schnittger I *et al*: Recommendations for quantitation of the left ventricle by two-dimensional echocardiography. American Society of Echocardiography Committee on Standards, Subcommittee on Quantitation of Two-Dimensional Echocardiograms. *J Am Soc Echocardiogr* 1989, 2(5):358-367.**

**4. Nagueh SF, Appleton CP, Gillebert TC, Marino PN, Oh JK, Smiseth OA, Waggoner AD, Flachskampf FA, Pellikka PA, Evangelista A: Recommendations for the evaluation of left ventricular diastolic function by echocardiography. *J Am Soc Echocardiogr* 2009, 22(2):107-133.**

**5. Moschietto S, Doyen D, Grech L, Dellamonica J, Hyvernat H, Bernardin G: Transthoracic Echocardiography with Doppler Tissue Imaging predicts weaning failure from mechanical ventilation: evolution of the left ventricle relaxation rate during a spontaneous breathing trial is the key factor in weaning outcome. *Crit Care* 2012, 16(3):R81.**

**Table S1 The parameters of the predictors of respiratory failure within 48 hours including NT-pro-BNP (after SBT)**

| Variables | OR | 95% CI | *P-*value | AUC |
| --- | --- | --- | --- | --- |
| Duration from onset to the first intubation | 1.185 | 1.018-1.381 | 0.029 | 0.686 |
| Hemoglobin | 1.070 | 1.020-1.123 | 0.006 | 0.689 |
| PaCO_2_ after SBT | 1.277 | 1.071-1.523 | 0.006 | 0.800 |
| NT-pro-BNP after SBT | 1.000 | 1.000-1.001 | 0.020 | 0.672 |

*AUC* area under curve, *CI* confidence interval, *NT-pro-BNP* N-terminal-pro-BNP, *PaCO_2_* arterial carbon dioxide tension, *OR* odds ratio, *SBT* spontaneous breathing trial.

**Table S2 The parameters of the predictors of re-intubation within 1 week in the extubation success subgroup**

| Variables | OR | 95% CI | *P-*value |
| --- | --- | --- | --- |
| PH after SBT | < 0.001 | 0.000-357.198 | 0.083 |
| DE (average) after SBT | 0.339 | 0.105-1.100 | 0.072 |

*CI* confidence interval, *DE* diaphragmatic excursion, *OR* odds ratio, *SBT* spontaneous breathing trial.

**Table S3 The D-RBSI in the re-intubation and non-intubation subgroups**

|  | Re-intubation within 1 week | | |  |
| --- | --- | --- | --- | --- |
| Variables | RI subgroup | NI subgroup | | *P* value |
| N (cases) | 10 | | 19 |  |
| **Before SBT** |  | |  |  |
| D-RSBI (RR/DE) | 2.71±1.67 | | 2.04±1.01 | 0.189 |
| **After SBT** |  | |  |  |
| D-RSBI (RR/DD) | 2.82±1.93 | | 1.49±0.61 | 0.060 |

*DE* diaphragmatic excursion, *D-RBSI* diaphragmatic-rapid shallow breathing index, *NI* non-intubation, *RI* re-intubation, *RR* respiratory rate, *SBT* spontaneous breathing trial.
